# The difference in postoperative pulmonary functional change between upper and lower thoracoscopic lobectomy

**DOI:** 10.1093/icvts/ivab268

**Published:** 2021-10-04

**Authors:** Shinya Tane, Mai Kitazume, Yusuke Fujibayashi, Sanae Kuroda, Kenji Kimura, Yoshitaka Kitamura, Daisuke Takenaka, Wataru Nishio

**Affiliations:** 1 Division of Chest Surgery, Hyogo Cancer Center, Akashi, Japan; 2 Division of Diagnostic Radiology, Hyogo Cancer Center, Akashi, Japan

**Keywords:** *D*-value, Lobectomy, Lung function, Thoracoscopy

## Abstract

**OBJECTIVES:**

Through 3-dimensional lung volumetric and morphological analyses, we aimed to evaluate the difference in postoperative functional changes between upper and lower thoracoscopic lobectomy.

**METHODS:**

A total of 145 lung cancer patients who underwent thoracoscopic upper lobectomy (UL) were matched with 145 patients with lung cancer who underwent thoracoscopic lower lobectomy (LL) between April 2012 and December 2018, based on their sex, age, smoking history, operation side, and pulmonary function. Spirometry and computed tomography were performed before and 6 months after the operation. In addition, the postoperative pulmonary function, volume and morphological changes between the 2 groups were compared.

**RESULTS:**

The rate of postoperative decreased and the ratio of actual to predicted postoperative forced expiratory volume in 1 s were significantly higher after LL than after UL (*P* < 0.001 for both). The tendency above was similar irrespective of the resected side. The postoperative actual volumes of the ipsilateral residual lobe and contralateral lung were larger than the preoperatively measured volumes in each side lobectomy. Moreover, the increased change was particularly remarkable in the middle lobe after right LL. The change in the *D*-value, representing the structural complexity of the lung, was better maintained in the left lung after LL than after UL (*P* = 0.042).

**CONCLUSIONS:**

Pulmonary function after thoracoscopic LL was superior to that after UL because the upward displacement and the pulmonary reserves of the remaining lobe appeared more robust after LL.

**Figure:**
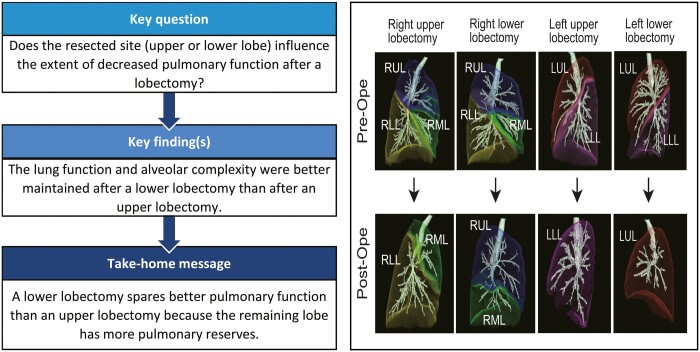

## INTRODUCTION

Segmentectomy has gained popularity among thoracic surgeons for small peripheral lesions. Nonetheless, lobectomy remains the ‘gold standard procedure’ for non-small-cell lung cancer [1]. However, this procedure causes a greater extent of pulmonary function loss than limited resection such as segmentectomy. This functional loss could particularly increase the burden on lobectomized patients with poor pulmonary function [2]. Thus, it is important to accurately predict the postoperative functional status after lobectomy.

The impact of the resected site (upper or lower lobe) on the extent of decreased pulmonary function remains controversial. Upper lobectomy (UL) has an increased effect on pulmonary function due to its volume reduction effect [3]. In contrast, previous studies have demonstrated that the decrease of lung function after UL is higher than that after lower lobectomy (LL) [4, 5]. The possible mechanisms could be that relatively larger lower lobe resection could promote a compensatory response of the remaining lung. Furthermore, the upper lobe probably has more alveolar-capillary reserves than the lower lobe that can be recruited postoperatively. However, the mechanisms mentioned above have not been proven pathologically or morphologically. Thus, we need to evaluate the difference in postoperative pulmonary function between UL and LL and to understand the underlying mechanism.

The *D*-value is generally used to evaluate the structural quality of the lungs [6]. Distributions of low-attenuation area (LAA) sizes follow an approximately straight line when plotted on a log–log graph and are considered a power of law. The slope of this distribution graph, which represents the *D*-value, tends to flatten with the progression of emphysema. This indicates that *D*-value reflects the complexity of the alveolar structure and facilitates the objective and comprehensive quantitative assessment of the lung tissue destruction in pulmonary emphysema [7]. Therefore, *D*-value can also be used to evaluate pulmonary morphological changes after resection.

In the current study, we aimed to evaluate the differences in postoperative functional changes between upper and lower thoracoscopic lobectomy and identify the morphological reaction after lobectomy of upper and lower lobes.

## MATERIALS AND METHODS

### Ethical statement

The Hyogo Cancer Center Institutional Review Board (IRB) approved this study (IRB number; G-102: approved on 26 November 2019), and each participant provided informed consent.

### Patients

A total of 497 consecutive patients with primary lung cancer who underwent thoracoscopic UL or LL at the Hyogo Cancer Center between April 2012 and December 2018 were examined.

A total of 145 patients who underwent thoracoscopic UL were matched with 145 patients who underwent thoracoscopic LL, based on their sex, age, smoking history, operation side and pulmonary function, using propensity score matching. In addition, computed tomography (CT) and spirometry were performed before and 6 months after the operation. Patients who received induction therapy, underwent bilobectomy or had a history of lung resections were excluded from our study cohort.

The operation was generally performed through 4 port sites (2–4 cm) without rib spreading and direct vision. We reviewed the postoperative pulmonary complications, age, sex, smoking history, body mass index, operation side, operation site, operation time, blood loss volume, forced expiratory volume in 1 s (FEV1.0) and forced vital capacity; FEV1.0 and forced vital capacity were measured by spirometry. Operative mortality was defined as death within 30 days of the resection.

### Computed tomography and 3-dimensional lung image construction

All plain chest CT examinations were performed using 16- or 80-multidetector row CT scanners (Aquilion 16 or Prime, Toshiba Medical Systems, Otawara, Japan). The whole lung was scanned from the lung apex to the diaphragm during a single breath-hold at end-inspiration. Scan parameters of the multidetector raw CT examination were as follows: 130 kVp; 150 mAs; collimation 1 mm × 16; rotation 0.5 s; or 120 kVp; 390–500 mAs; collimation 0.5 mm × 80; and rotation 0.35 s; 512 × 512 matrix; Field of view 320 mm; reconstruction 1 mm/1 mm.

Three-dimensional imaging was reconstructed from the CT data using the Synapse Vincent software program (Fujifilm Corp, Tokyo, Japan). This software enables the measurement of the volume of the resected lobe, the ipsilateral unaffected lobe and the contralateral lung using preoperative CT data. These volumes after thoracoscopic lobectomy were also calculated from the postoperative CT data.

### Pulmonary functional analysis

Predicted postoperative (PPO) FEV1.0 after lobectomy was calculated using the following formula:

Preoperative FEV1.0 × (residual lung volume after lobectomy/whole lung volume measured from preoperative 3D CT).

The percentage of actual postoperative/PPO FEV1.0 after lobectomy was defined as APO/PPO FEV1.0.

### Image interpretation and morphological analysis

**Figure 1:**
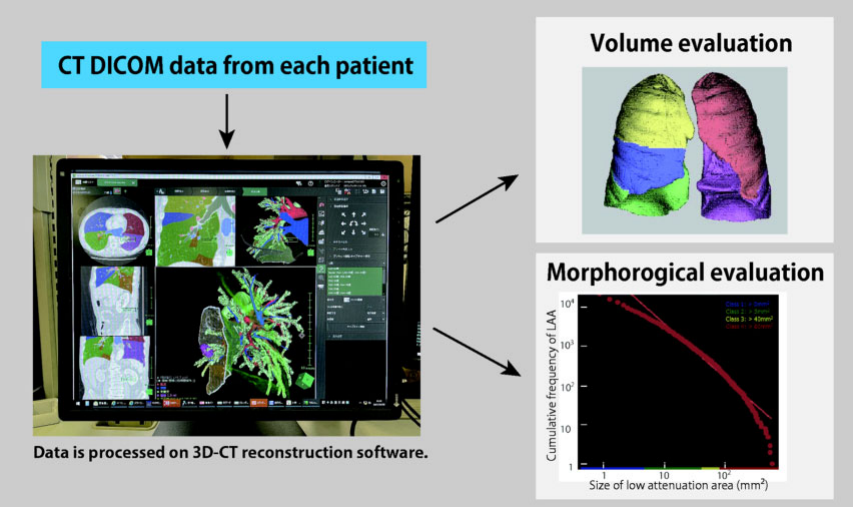
Volumetric and morphological analyses on the 3D CT software. 3D CT: 3-dimensional computed tomography; DICOM: Digital Imaging and Communications in Medicine; LAA: low-attenuation area.

As illustrated in Fig. 1, LAA and *D*-value according to LAA size, representing morphological parameter, can be obtained using the SYNAPSE VINCENT. LAA was defined as lung area ≤−950 Hounsfield units. The cumulative frequency-size distribution of the LAA supposedly followed a power-law characterized by an exponent *D*, as shown below:

$$Y=\mathrm{KX}{‒}^{\text{D}}$$

where *X* is the size of the LAA, *Y* is the cumulative frequency and *K* is a constant. Using the SYNAPSE VINCENT, the values of *D* were obtained by linear regression and calculated as the slope of the straight line on log-log plots. A higher *D*-value indicates maintenance of complexity of the alveolar structure. In contrast, a lower *D*-value indicates an increased prevalence of large, low-attenuation clusters, suggesting the destruction of the alveolar tissue.

### Statistical analyses

To compare the UL with LL outcomes, we used propensity score matching based on age, sex, smoking history, resected side and preoperative pulmonary function, including FEV1.0 and FEV1.0/forced vital capacity. Moreover, this helped us to reduce bias. Matching was performed at a ratio and calliper distance of 1:1 and 0.05, respectively. Match balance between the groups was assessed with the standardized mean differences of all variables included in the propensity score estimation and was considered appropriate if none of the standardized mean differences exceeded 0.1. The Pearson’s *χ*^2^ test or Fisher’s exact test was used for categorical comparison of complications and clinical factors, and the Student’s *t*-test or Wilcoxon rank-sum test was used for continuous data, depending on the normality of distribution. Various parameters, including the percentage of preoperative/postoperative and APO/PPO FEV1.0, and change in *D*-value between UL and LL, were compared using a paired *t*-test. All tests were two-sided, and *P*-values <0.05 indicated a significant difference.

All statistical analyses were performed using the JMP software program (version 13, SAS Inc., Cary, NC, USA). All values were expressed as mean ± standard deviation.

## RESULTS

### Operative results

Patient characteristics and results of the overall operation are summarized in Table 1. The average operation time and amount of bleeding were 187 min and 66 ml and 182 min and 61 ml in UL and LL, respectively. Moreover, there were no significant differences in the operation time and blood loss volume between UL and LL groups (*P* = 0.85 and *P* = 0.56, respectively). There were 28 (19.3%) and 24 (16.5%) cases of postoperative complications in the UL and LL groups, respectively. In addition, the complication rate was not significantly different between the groups (*P* = 0.54).

**Table 1:** Summary of the clinical variables before and after propensity score matching analysis

Clinical variables	Before propensity score matching				After propensity score matching			
	UL (*n* = 302)	LL (*n* = 195)	*P*-value	SMD	UL (*n* = 145)	LL (*n* = 145)	*P*-value	SMD
Sex (M/F)	175/127	104/91	0.34	0.092	75/70	76/69	0.48	0.001
Age (years)	68.7 ± 9.5	69.4 ± 9.6	0.37	0.001	69.0 ± 9.5	68.8 ± 9.3	0.51	0.001
Side (right/left)	227/75	130/65	0.041	0.18	109/36	109/36	1	0.001
Smoking history (yes/no)	177/125	112/83	0.79	0.15	78/67	80/65	0.63	0.018
FEV1.0 (l)	2.32 ± 0.61	2.21 ± 0.58	0.058	0.29	2.24 ± 0.07	2.25 ± 0.07	0.56	0.081
FEV1.0/FVC (l)	75.2 ± 9.7	74.3 ± 10.4	0.33	0.001	74.4 ± 10.7	74.3 ± 8.3	0.84	0.001
Operation time (min)	180 ± 42.0	189 ± 42.2	0.027		182 ± 44.9	187 ± 47.8	0.87	
Blood loss (ml)	60.8 ± 56.8	66.4 ± 60.2	0.32		51.4 ± 44	59.3 ± 49	0.56	
Postoperative complications (%)	56 (18.5)	34 (17.4)	0.75		28 (19.3)	24 (16.5)	0.54	

FEV1.0: forced expiratory volume in 1 s; FVC: forced vital capacity; LL: lower lobectomy; UL: upper lobectomy; SMD: standard mean difference.

There were no instances of postoperative deaths.

### Comparison of the postoperative pulmonary function between upper and lower lobectomy

**Figure 2:**
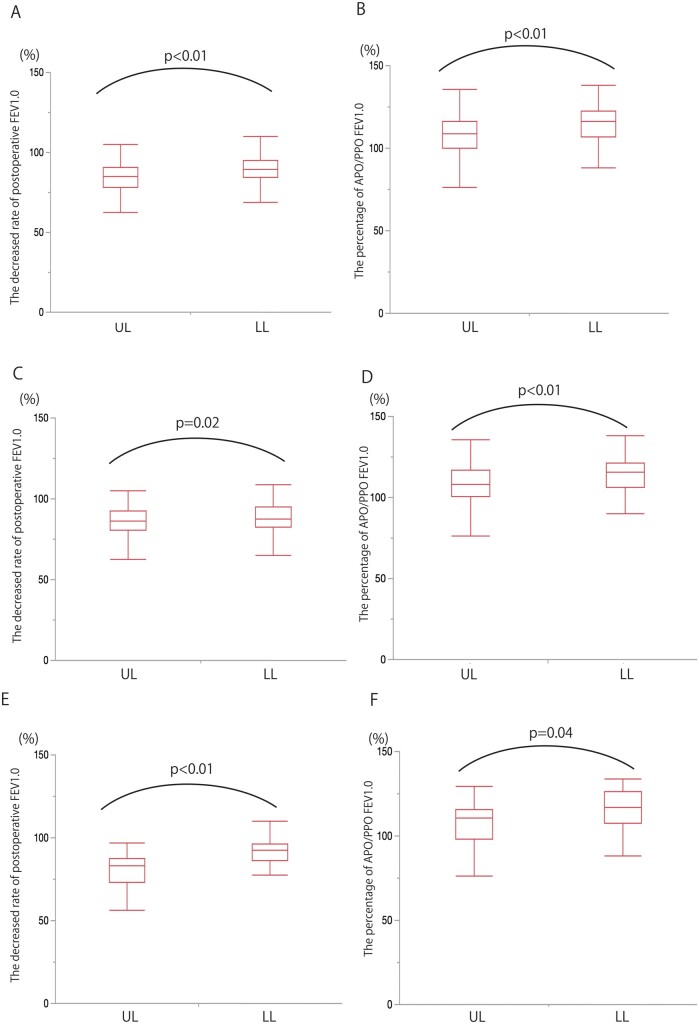
A comparison of the decreased rate of postoperative FEV1.0 and the APO/PPO FEV1.0 between UL and LL (**A and B**). The parameters were separately compared on the right (**C and D**) and left sides (**E and F**). APO/PPO FEV1.0; Actual/predictive postoperative forced expiratory volume in 1 s; LL: lower lobectomy; UL: upper lobectomy.

Overall, the decreased rate of postoperative FEV1.0 was significantly higher after LL than after UL (90% vs 83%, *P* < 0.001) (Fig. 2A). Moreover, the APO/PPO FEV1.0 was significantly higher after LL than after UL (115% vs 107%, *P* < 0.001) (Fig. 2B).

Next, we examined the postoperative pulmonary function based on the resected side. The decreased rate of postoperative FEV1.0 was better preserved in the right lung after LL than that after UL (88% vs 84%, *P* = 0.025) (Fig. 2C). In addition, the APO/PPO FEV1.0 was significantly higher after LL than after UL (114% vs 106%, *P* = 0.002) (Fig. 2D).

Likewise, in the left lung, the decreased rate of postoperative FEV1.0 and APO/PPO FEV1.0 was higher after LL than after UL (94% vs 81%, *P* < 0.001; 118% vs 108%, *P* = 0.043, respectively) (Fig. 2E and F). Therefore, thoracoscopic LL preserved the postoperative pulmonary function better than UL, irrespective of the resected side.

### Lung volumetric analysis

**Figure 3:**
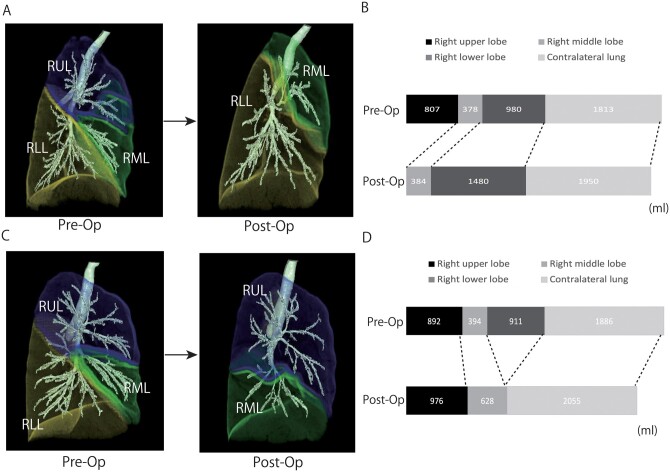
Changes in the lung volume in each lobe before and after RUL (**A and B**) or RLL (**C and D**). The ipsilateral residual lobe and contralateral lung volumes after lobectomy were larger than those before lobectomy in each procedure, and the changes were more remarkable in the middle lobe after lower lobe. RLL: right lower lobe; RML: right middle lobe; RUL: right upper lobe.

**Figure 4:**
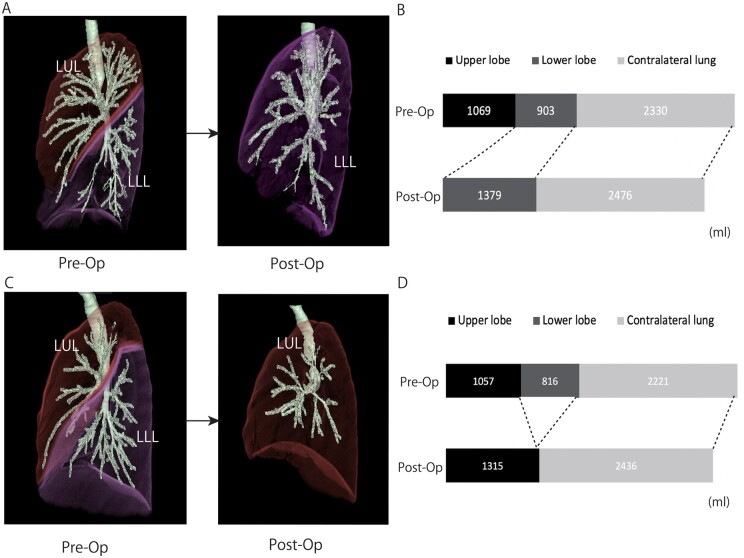
Changes in the lung volume in each lobe before and after LUL (**A and B**) or LLL (**C and D**). Overall, the ipsilateral residual lobe and contralateral lung volumes after lobectomy were larger than those before lobectomy in each procedure. LLL: left lower lobe; LUL: left upper lobe.

The dynamic changes in lung volumes before and after lobectomy were examined (Figs 3 and 4). Overall, volumes of the ipsilateral remaining lobe and contralateral lung increased after lobectomy on each side, indicating an over inflation of the remaining lungs. Particularly in the right lung, the lobe volume of the middle lobe significantly increased after LL, compared to that after UL (*P* < 0.001). Our results could be explained by the phenomenon of an upward displacement of the middle lobe after removal of the upper lobe that induces a marked depression of the ipsilateral residual lung.

### Morphological analysis

We examined the structural reaction after left side lobectomy in each lobe to understand why LL spares postoperative pulmonary function better than UL. Table 2 summarizes the *D*-values of the remaining ipsilateral lobe, before and after lobectomy. The *D*-value of the upper lobe was maintained after LL, while that of the lower lobe was significantly decreased after UL (*P* = 0.002). In addition, the change in *D*-value in the ipsilateral residual lung was significantly greater after UL than that after LL (*P* = 0.042). This indicated that the alveolar complexity in the ipsilateral lung was better maintained after LL than after UL. Therefore, we assumed that the upper lobe might have more alveolar-capillary reserves than the lower lobe to be recruited postoperatively.

**Table 2:** Changes in *D*-values before and after lobectomy

	LUL		LLL	
	Before lobectomy	After lobectomy	Before lobectomy	After lobectomy
*D*-value	2.64 ± 1.17	2.16 ± 0.93	2.44 ± 0.91	2.36 ± 0.89
Changes in *D*-value	87 ± 3 (%)		98 ± 4 %	

LLL: left lower lobectomy; LUL: left upper lobectomy.

## DISCUSSION

The pulmonary function was well maintained after LL compared to that after UL in all the procedures performed uniformly through a thoracoscopic approach. The novelty of this study was that it evaluated postoperative pulmonary function through volumetric and morphological analyses based on the cutting-edge 3D-reconstruction software. Several studies have reported that LL preserves postoperative pulmonary function better than UL, which is consistent with our findings [4, 5]. The studies mentioned above have not clearly explained the difference in postoperative lung function between UL and LL. However, researchers have proposed several possible explanations; (i) following UL, the bronchial angle through the upward displacement of the remaining lung can lead to deformity of the residual bronchus, resulting in decreased postoperative lung function; (ii) impact of LL on the compensatory response was greater than that of UL because relatively larger lung resection supposedly promotes the compensatory response of the remaining lung. However, the 3-dimensional CT measured volume of the lower lobe was smaller than that of the upper lobe. Therefore, we hypothesized that the upper lobe possibly has more alveolar-capillary reserves than the lower lobe to be recruited postoperatively, in addition to the reasons mentioned above.

We adopted *D*-value as a measure of the pulmonary structural quality after lobectomy. The *D*-value has been used since the 1990s for evaluating pulmonary emphysema. Mishima *et al.* [6] first applied the method of fractal geometry, developed by Mandelbrot, to quantify pulmonary emphysema. The cumulative frequency-size distribution of low-attenuation lung regions followed a power-law characterized by an exponent *D*. There has been increasing evidence on the association between *D*-value and the progression of chronic obstructive pulmonary disease [8, 9]. *D*-value likely predicts pulmonary complications after thoracoscopic lobectomy [10]. In addition to the quantitative assessment of pulmonary emphysema, the *D*-value has been utilized to evaluate the structural quality of the lungs [11]. We employed the concept of *D*-value to compare the structural changes between the upper and lower lobes after lobectomy.

The alveolar complexity of the ipsilateral residual lobe was well maintained after LL. Moreover, the pulmonary reserves appeared more robust in the upper lobe. This potential reserve of the upper lobe is presumably associated with the thoracic motion. Jahani *et al.* [12] assessed the regional ventilation of healthy human lungs using 4-dimensional CT and suggested that the lower lobes always contribute more to changes in the air volume than the upper and middle lobes during dynamic breathing. Yilmaz *et al.* [13] examined progressive adaptation in the regional parenchyma mechanics following lung resection by using functional CT on a canine model. They demonstrated that regional displacement was most pronounced in the caudal region. Therefore, we speculate that following LL, the remnant lungs reach a stage of contact with diaphragm motion. This eventually recruits the pulmonary reserves postoperatively, leading to greater recovery in the upper lobes.

UL possibly causes dystelectasis of the remaining lower lobe because of the upward displacement of the remaining lung. In addition, there are reports on middle lobe dystelectasis, particularly after right UL. This can be attributed to the impact of anatomical deviation of the right middle lobar bronchus on the mechanism of pulmonary aeration [14]. The phenomenon above is in line with our results, demonstrating that the lobe volume of the middle lobe increases after LL and decreases after UL. Moreover, our findings were consistent with the recovery of the lingula after upper-division segmentectomy in the left lung. We previously demonstrated that the residual lobe (lingular) volume after upper-division segmentectomy was significantly lower than that expected preoperatively [15].

### Limitations 

The present study had several limitations. First, we compared the morphological changes between UL and LL only in the left lung. This was because middle lobe dystelectasis after UL could influence the morphological changes in the right lung. Second, we only examined the postoperative pulmonary function by calculating the lung volume without evaluating the perfusion of the residual lungs. Additional modalities, such as lung perfusion single-photon emission CT, could be considered for this purpose. In addition, other measurements such as diffusing capacity for carbon monoxide and quality of life were not compared. Third, we evaluated postoperative lung function only at 6 months after lung resection. A chronological evaluation at later points such as 1 or 5 years postoperatively will be needed to comprehend long-term functional change. Fourth, this was a non-randomized, retrospective, single-institutional study. Despite conducting a propensity score matching and performing all procedures uniformly through a thoracoscopic approach, the study design mentioned above is associated with patient selection and other biases.

## CONCLUSION

Postoperative lung function after thoracoscopic LL was superior to that after UL. However, the upward displacement of the remaining lobe after UL, particularly the middle lobe after right UL, could cause dystelectasis of the remaining lobe. In addition, the pulmonary reserves of the remaining lobe after LL appeared more robust through morphological evaluation.

## ABBREVIATIONS

APO: Actual postoperative
CT: Computed tomography
FEV1.0: Forced expiratory volume in 1 s
LAA: Low-attenuation area
LL: Lower lobectomy
PPO: Predicted postoperative
UL: Upper lobectomy
